# Clinical utility of diffusion MRI‐derived measures of cortical microstructure in a real‐world memory clinic setting

**DOI:** 10.1002/acn3.52097

**Published:** 2024-07-24

**Authors:** Mario Torso, Giorgio Fumagalli, Gerard R. Ridgway, Valeria Elisa Contarino, Ian Hardingham, Elio Scarpini, Daniela Galimberti, Steven A. Chance, Andrea Arighi

**Affiliations:** ^1^ Oxford Brain Diagnostics Ltd Oxford UK; ^2^ Center For Mind/Brain Sciences‐CIMeC University of Trento Rovereto Italy; ^3^ Neuroradiology Unit Fondazione IRCCS Ca’ Granda Ospedale Maggiore Policlinico Milan Italy; ^4^ Neurodegenerative Disease Unit Fondazione IRCCS Ca’ Granda Ospedale Maggiore Policlinico Milan Italy; ^5^ Dept. of Biomedical, Surgical and Dental Sciences University of Milan Milan Italy

## Abstract

**Objective:**

To investigate cortical microstructural measures from diffusion MRI as “neurodegeneration” markers that could improve prognostic accuracy in mild cognitive impairment (MCI).

**Methods:**

The prognostic power of Amyloid/Tau/Neurodegeneration (ATN) biomarkers to predict progression from MCI to AD or non‐AD dementia was investigated. Ninety patients underwent clinical evaluation (follow‐up interval 32 ± 18 months), lumbar puncture, and MRI. Participants were grouped by clinical stage and cerebrospinal fluid Amyloid and Tau status.

T1‐structural and diffusion MRI scans were analyzed to calculate diffusion metrics related to cortical columnar structure (AngleR, ParlPD, PerpPD^+^), cortical mean diffusivity, and fractional anisotropy. Statistical tests were corrected for multiple comparisons. Prognostic power was assessed using receiver operating characteristic (ROC) analysis and related indices.

**Results:**

A progressive increase of whole‐brain cortical diffusion values was observed along the AD continuum, with all A+ groups showing significantly higher AngleR than A−T−.

Investigating clinical progression to dementia, the AT biomarkers together showed good positive predictive value (with 90.91% of MCI A+T+ converting to dementia) but poor negative predictive value (with 40% of MCI A−T− progressing to a mix of AD and non‐AD dementias). Adding whole‐brain AngleR as an N marker, produced good differentiation between stable and converting MCI A−T− patients (0.8 area under ROC curve) and substantially improved negative predictive value (+21.25%).

**Interpretation:**

Results support the clinical utility of cortical microstructure to aid prognosis, especially in A−T− patients. Further work will investigate other complexities of the real‐world clinical setting, including A−T+ groups. Diffusion MRI measures of neurodegeneration may complement fluid AT markers to support clinical decision‐making.

## Introduction

Dementia represents one of the main medical problems and arises from a variety of neuropathological processes and injuries that primarily or secondarily affect the human brain. These brain changes start many years before clinical onset,^1^ so a timely and accurate diagnosis plays a key role, enabling therapeutic decisions and support for individuals.

In the last decade, the combination of fluid amyloid beta (Aβ42, Aβ40, Aβ 42/40 ratio), phosphorylated tau (pTau‐181, pTau‐217, etc.) biomarkers, and magnetic resonance imaging (MRI) have been shown to have predictive value for progression to dementia.^2^, ^3^, ^4^ For Alzheimer's disease (AD), a biological framework for diagnosis, based on the presence of pathology rather than the presence of clinical symptoms, has been proposed^5^ and is currently being updated. The Amyloid/Tau/Neurodegeneration (ATN) framework^5^ is well‐established in research, with biomarkers seen as crucial for an accurate diagnosis. Revision of the ATN framework is under way, with current discussion around how to develop the framework for clinical use (https://aaic.alz.org/diagnostic‐criteria.asp). While biomarkers are widely used in clinical AD research, in routine clinical practice, doubts remain about potential overdiagnosis or missed diagnoses, differential diagnosis, and the best way to operationalize the framework.

The current guidelines and consensus documents supporting the diagnostic use of biomarkers in clinical practice suggest that a combination of different fluid and imaging biomarkers can improve the diagnostic pathway.^6^ However, there is significant variability across memory clinics in the availability, frequency of use, and confidence in the diagnostic utility of these markers.^7^, ^8^ In addition, in a real‐world clinical setting, a lot of patients can show early symptoms overlapping with early AD, but caused by different underlying non‐AD neuropathological processes, as confirmed by amyloid and tau biomarker negativity, many of which do not still have robust and specific in vivo biomarkers.^5^ Considering that even the non‐AD neuropathological processes determine neurodegeneration (N) (e.g., TDP‐43 and alpha‐synuclein), a useful support to diagnosis can be provided by neurodegenerative markers. In addition, the N biomarker group provides important pathologic staging information^5^ and can be useful for differential diagnosis.

Neurofilament light chain (NfL) is one of the most promising fluid biomarkers but is a nonspecific marker for AD neuronal injury, being increased in AD and non‐AD diseases, such as frontotemporal dementia, amyotrophic lateral sclerosis, and atypical Parkinson's disorder,^9^ and it does not provide any information about the brain regions damaged. A recent study^10^ investigated the added value of NfL, to the existing diagnostic methods and biomarkers, in a mixed memory clinic cohort of consecutive patients, showing that, while cerebrospinal fluid (CSF) NfL led to increased diagnostic certainty for the specialist in neurology, it did not increase the diagnostic accuracy of the etiological diagnoses. The conventional structural MRI and fluorodeoxyglucose positron emission tomography (FDG‐PET) are often not very useful for differential diagnosis in the early stages of the disease, due to a lack of clear atrophic/hypometabolic signature in the mild cognitive impairment (MCI) stage or earlier,^11^, ^12^ and discordances between imaging and CSF biomarkers that may occur.^5^ Therefore, the diagnosis and prognosis of clinically impaired patients (MCI) without clear structural imaging evidence of neurodegeneration and in the absence of any significant amyloid and tau accumulation (A−T−N−) represents a frequent challenge on clinical grounds.

In recent years, cortical diffusivity measurements have been proposed to investigate early microstructural cortical changes and as potential imaging markers of neurodegeneration.^13^, ^14^, ^15^, ^16^, ^17^

Previous studies have shown that cortical mean diffusivity (MD)^16^, ^17^ and a novel set of columnar‐related measures^13^, ^14^ are sensitive to cortical microstructural alterations and associated with the main AD neuropathological hallmarks, such as Thal phase, Braak staging, and AD neuropathological changes (ADNC) “ABC” score.^15^

The main aims of the present study were to investigate cortical microstructural alterations across the amyloid continuum (A−T−, A+T−, A+T+) in participants with clinical symptoms (MCI and dementias) and to test cortical diffusivity measures as sensitive N markers and a potential way to improve diagnostic accuracy in patients with MCI A−T−.

To better investigate the diagnostic capacity of N markers, we have decided to focus our attention on the two extremes of the amyloid continuum (A−T− and A+T+).

In the A−T− group, we expect that negativity to N markers could confirm the absence of AD and other forms of cortical neurodegeneration, while positivity could indicate the presence of non‐AD processes (not measurable with CSF markers of amyloid or tau).

In the A+T+ group, positivity to N markers could confirm the presence of neurodegeneration due to the deposition of amyloid and tau, while negativity could indicate a relative preservation of cortical architecture.

We note that the A+T− group is complex and could be a confounding factor for investigations into diagnostic power, as it may indicate an early stage of Alzheimer's progression (amyloid positivity but not yet tau) or the positivity to amyloid might be a comorbidity (secondary) to another neuropathology not measurable with AD biomarkers (e.g., TDP‐43).

We hypothesized that since neurodegenerative biomarkers reveal complementary information, a combination of CSF and cortical microstructural MRI biomarkers may increase the diagnostic accuracy.

## Methods

### Participants

Patients suspected to have dementia without structural abnormalities or additional neurological disorders (e.g., stroke) previously recruited at the Neurodegenerative Diseases Unit of the Fondazione Ca′ Granda, IRCCS Ospedale Maggiore Policlinico (Milan, Italy) between January 2015 and June 2019 were included in the study.

All participants underwent neurological and neuropsychological examinations, including the Mini‐Mental State Examination (MMSE), MRI scanning, and lumbar puncture for the determination of AD biomarkers.

The patients were diagnosed by expert neurologists with MCI^18^ or dementia based on the current criteria. Participants with AD met the diagnostic criteria for AD as defined by National Institute on Aging‐Alzheimer's Association workgroups on diagnostic guidelines for AD.^19^, ^20^ Participants with non‐AD dementia were diagnosed according to the specific criteria of each other clinical syndrome.^21^, ^22^, ^23^, ^24^, ^25^, ^26^

Levels of Aβ42, total tau (tTau), and phosphorylated tau 181(pTau‐181) were measured from CSF samples using Innotest ELISAs following the manufacturer's instructions (Fujirebio, Ghent, Belgium). For the purpose of the study, participants were classified for Aβ42 (A+ or A−) and pTau‐181 (T+ or T−) using the local laboratory thresholds of positivity as used in earlier publications: Aβ42 ≤ 600 pg/mL^27^, ^28^; pTau‐181 ≥ 61 pg/mL.^29^ These thresholds were obtained by consideration of the receiver operating characteristic (ROC) curve and selection of the point that maximized the Youden index.

The diagram shown in Figure 3 summarizes the process of participant inclusion in the study. Out of a total of 124 available scans, 16 were excluded due to missing or incomplete CSF values. Additionally, 18 scans were excluded because they exhibited a CSF A−T+ profile. No scans were excluded due to artifacts or structural anomalies.

Ninety participants were eligible for the study according to the following criteria: (1) diagnosis of MCI or dementia^18^; (2) availability of 3T MRI scans, including the three‐dimensional (3D) volumetric T1‐weighted and diffusion MRI; (3) availability of Aβ42 (A) and pTau‐181 (T) CSF measures; and (4) CSF AT profile A−T−, A+T−, and A+T+.

Based on clinical syndromic status and CSF Aβ42 (A) and pTAU‐181 (T) status, the participants were further grouped into MCI A−T−, MCI A+T−, MCI A+T+, Dementia A+T−, and Dementia A+T+.

All procedures performed in the study were in accordance with the ethical standards of the institutional and/or national research committee and with the 1964 Helsinki Declaration and its later amendments or comparable ethical standards.

This retrospective study was approved by the Ethical Committee of the Fondazione IRCCS Ca′ Granda Ospedale Maggiore Policlinico (Comitato Etico Area 2 Milano, approval N 859_2021, date 14 September 2021). Informed consent was obtained from all the participants.

### MRI acquisition

The MRI was performed with a 3‐tesla scanner (Achieva, Philips Healthcare, Eindhoven, Netherlands) using a 32‐channel phased‐array head coil. Whole‐brain 3D T1‐weighted turbo field‐echo sequence images were acquired in the sagittal plane with the following parameters: repetition time (TR) = 9.8 ms, echo time (TE) = 4.6 ms, flip angle = 8°, matrix = 256 × 256, and voxel size = 0.94 × 0.94 × 1 mm^3^. DTI data were acquired using two protocols, each with voxel size = 2 × 2 × 2 mm, matrix = 112 × 112, b‐value = 1000s/mm^2^, but one with 32 gradient directions, TR = 9966.4 ms; TE = 55 ms; and the second one with 64 gradient directions, TR = 3940 ms, TE = 74 ms. The protocol change was due to a machine upgrade. The two protocols were not harmonised before and after the upgrade; instead we adjust statistically for potential differences between the protocols with a factor in the general linear model, described below.

### Structural MRI analyses

The 3D T1‐weighted images for each participant were segmented using FreeSurfer v 6.0 (https://surfer.nmr.mgh.harvard.edu/)^30^ and used to assess the cortical gray matter volume, hippocampal volume, and the cortical thickness.

The two hippocampal volumes obtained (left and right) were averaged. To account for participants' head size differences, volumes were expressed as a percentage of the total intracranial volume, namely cortical volume fraction (CVF) and bilateral hippocampal volume fraction (HVF).

### Cortical diffusivity analysis

T1 structural and diffusion tensor imaging (DTI) scans were combined to perform cortical diffusivity analysis.

Diffusion‐weighted images were preprocessed using FSL tools (FSL Version 6.0; FMRIB Software Library, Oxford, UK – https://www.fmrib.ox.ac.uk/fsl/). Diffusion‐weighted images were corrected for motion and eddy current effects^31^ by alignment of all images to a reference b = 0 image using FSL's eddy tool. The diffusion tensor was then calculated with the FSL DTIFIT tool, providing fractional anisotropy (FA), mean diffusivity (MD), etc. For each participant, the displacement among diffusion volumes was estimated using the eddy output to obtain a measure of head motion during the acquisition.

**Figure 1:**
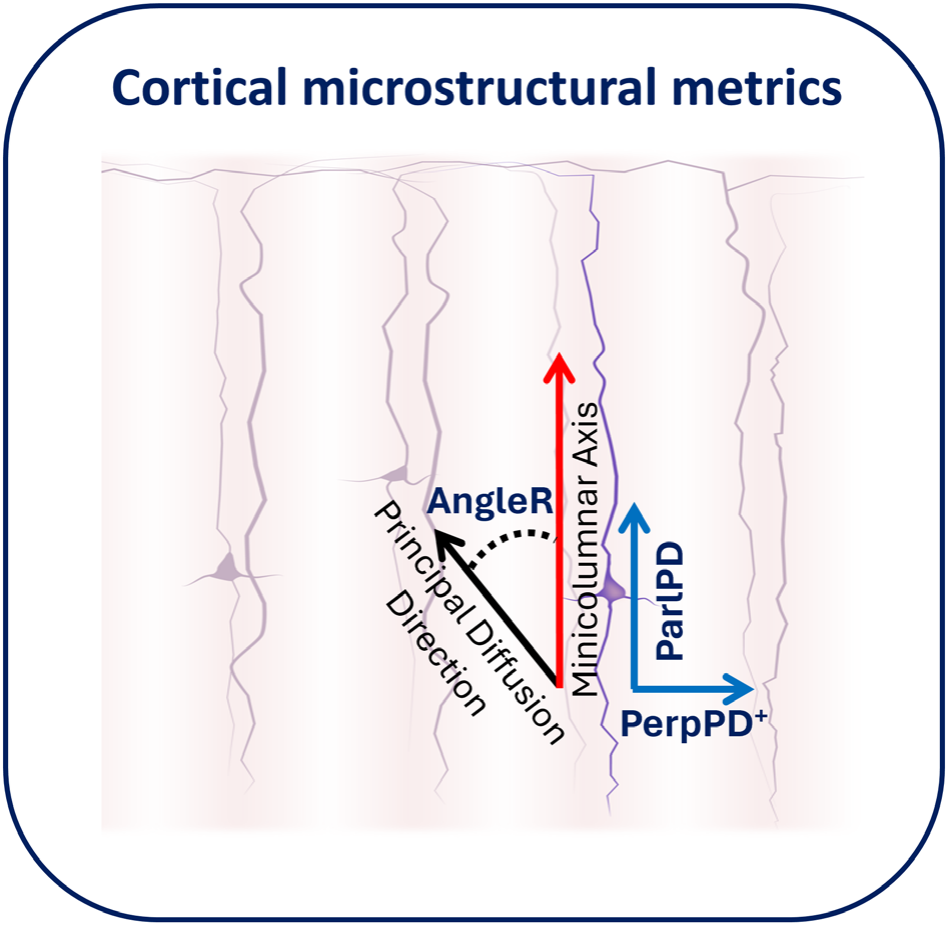
Cortical microstructural metrics. This figure shows a cross‐sectional image of diffusion based metrics related to the three directional components of diffusion within the cortical gray matter.

Standard diffusivity analysis was conducted to calculate FA and MD in the cortex (restricted to relatively pure cortical tissue with high confidence of being GM). Further cortical diffusivity analysis was performed using a proprietary software tool (patent WO2016162682A1). The tool generates cortical profiles, providing an estimate of the columnar axis within the cortex. Values for the diffusion tensor derived metrics were averaged along the cortical profiles, throughout cortical GM.^13^, ^14^, ^32^ Briefly, three measures were calculated, relating to the components of diffusion (Fig. 1): AngleR was the angle between the radial minicolumn axis and the principal diffusion direction (in radians); ParlPD was the principal diffusion component parallel with the radial minicolumns (×10^−3^ mm^2^/s) and PerpPD^+^ combined the components perpendicular to the radial minicolumns (×10^−3^ mm^2^/s).

### Statistical analyses

Statistical analyses were performed using SPSS software (version 29.0; SPSS Inc., Chicago IL, USA). Demographic and clinical values were investigated using chi‐square tests for categorical measures and analysis of variance (ANOVA) tests for continuous values.

Group differences in cortical micro‐ and macrostructure were identified by general linear model (GLM) statistical analysis with group ID (A−T−, A+T− and A+T+), acquisition protocol (a binary factor for the two DTI protocols), and sex as fixed factors, with age and head motion as covariates.

Pairwise comparisons between groups were performed using the estimated marginal means and post hoc Bonferroni adjusted.

The discrimination power of the AT CSF biomarkers in MCI A−T− vs MCI A+T+ groups, based on predefined cutoffs, was investigated using conventional indices (sensitivity, specificity, accuracy, positive and negative likelihood, positive and negative predictive values). Mixed AT profiles (A−T+ or A+T−) were excluded, in order to reduce the potential for including inaccurately classified cases.

The test was conducted in the MCI A−T− and A+T+ groups, by comparing against the clinical reference standard (MCI clinical status: Converted or Stable). The expected profile for MCI Converted was A+T+, and the expected profile for MCI Stable was A−T−.

The discrimination power of the MRI N biomarkers in MCI A−T− groups was assessed by receiver operating characteristic (ROC) curve analysis and conventional indices. Every measure entered into the ROC analysis was adjusted for sex, age, acquisition protocol, and head movement. To establish the presence or absence of the target condition, we utilized the clinical status (MCI stable or MCI converted to dementia) as the reference standard.

The clinical diagnoses were made previously, so MRI metrics used in the present study were not available to the clinicians. Conversely, the extraction of MRI values was entirely automatic, and hence not influenced by the clinical diagnoses.

## Results

### Participants

The demographic and clinical characteristics of the sample are presented in Table 1. According to the CSF Aβ42 and pTau‐181 positivity, 25 participants were classified as A−T−, 28 as A+T−, and 37 as A+T+. To focus our investigations on the amyloid continuum, participants with a suspected non‐Alzheimer disease pathophysiology (SNAP) as showed by CSF biomarkers (A−T+) were not included in the study. No significant difference in age and years of formal education among the three groups were detected, while the A+T+ group showed a significantly different sex distribution compared with A−T− (*χ* ^2^ = 5.003; *p* < 0.05) and A+T− (*χ* ^2^ = 7.2637; *p* < 0.05) groups.

**Table 1:** Demographics and clinical characteristics of the sample.

	A−T− *n* = 25	A+T− *n* = 28	A+T+ *n* = 37	*p*‐value
Age mean (SD)	69.05 (8.3)	69.13 (9.1)	71.58 (6.7)	n.s
Sex M/F	17/8*	21/8*	14/22	<0.05
Education mean (SD) years	11.2 (3.9)	9.9 (4.3)	10.9 (4.9)	n.s
MMSE mean (SD)	26.3 (2.4)* ^,^ ^#^	20.8 (5.5)	20.0 (6.9)	<0.005

MMSE, mini‐mental state examination. Significant differences were assessed using chi‐square tests for categorical measures and ANOVA for continuous measures.

* Significantly different compared with A+T−;

^#^ Significantly different compared with A+T+.

Compared with the A−T− group, the MMSE scores were significantly lower in A+T− and A+T+ groups (*F*_2,78_ = 7.047 *p* < 0.005).

### Whole‐brain micro‐ and macrostructural MRI in AT cohorts

**Figure 2:**
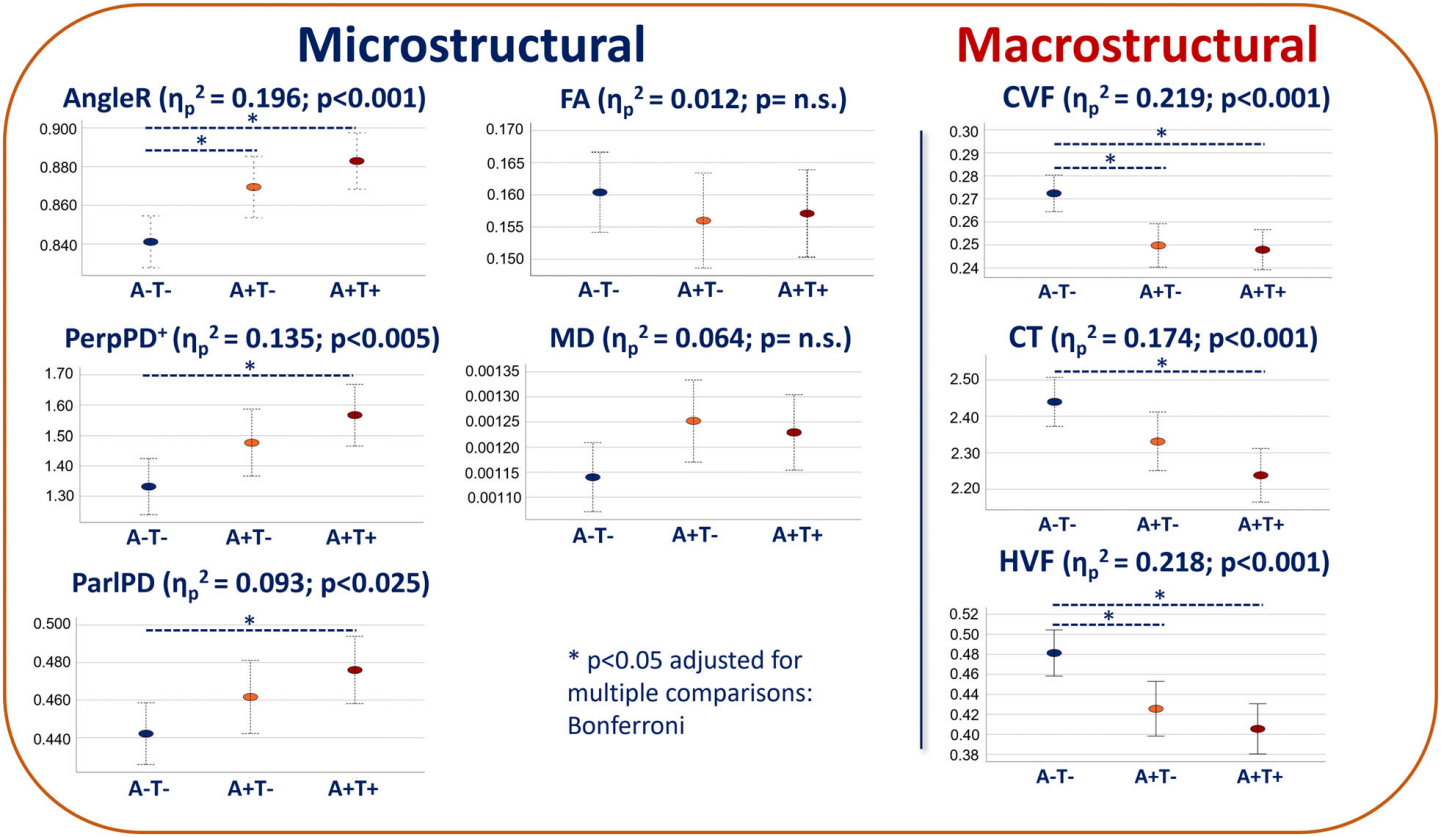
Micro‐ and macrostructural group differences. This figure shows micro‐ and macrostructural values across the AT continuum that includes MCI and Dementia groups. CVF, cortical volume fraction; CT, cortical thickness; HVF, hippocampal volume fraction.

Figure 2 summarizes micro‐ and macrostructural data. At the microstructural level, the cortical diffusivity metrics revealed higher values in both A+ groups than in A−T− group. The GLM revealed a significant effect of group on AngleR, PerpPD^+^, and ParlPD. AngleR was the only microstructural measure able to differentiate significantly A−T− and the other two studied groups (Table S1 Supplemental Material). No significant difference in whole‐brain FA and MD was observed between groups.

At the macrostructural level, the GLM showed a significant effect of group on all the measures investigated. Cortical volume fraction and HVF were able to differentiate between A−T− and both A+ groups.

No significant differences in micro‐ or macrostructural measures were observed comparing A+T− and A+T+ groups (for more information, see Table S1 Supplemental Material).

### Biological and syndromic diagnosis

Combining AT profile and clinical diagnosis, participants were subdivided into five different groups MCI A−T− (*n* = 25), MCI A+T− (*n* = 15), MCI A+T+ (11), Dementia A+T− (*n* = 13), and Dementia A+T+ (*n* = 26).

The demographic and clinical characteristics of the five studied subgroups are presented in Table 2. No differences in age and education were detected between groups. The Dementia A+T+ group showed a significantly different sex distribution compared with MCI A−T− (*χ* ^2^ = 5.6841; *p* < 0.05), MCI A+T− (*χ* ^2^ = 5.2637; *p* < 0.05), and Dementia A+T− (*χ* ^2^ = 4.1786; *p* < 0.05) groups.

As expected, MMSE score in the MCI groups was higher than that in the dementia groups (*F*_2,78_ = 18.597 *p* < 0.001).

**Table 2:** Summary characteristics of participants.

	MCI A−T− *n* = 25	MCI A+T− *n* = 15	MCI A+T+ *n* = 11	Dementia A+T− *n* = 13	Dementia A+T+ *n* = 26
Age mean (SD)	69.05 (8.3)	72.84 (8.4)	73.46 (5.3)	65.36 (8.3)	70.43 (7.2)
Sex (M/F)	17/8*	11/4*	6/5	9/4*	9/17
Education mean (SD) years	11.2 (3.9)	11.6 (4.0)	12.4 (5.1)	8.3 (3.9)	10.1 (4.9)
MMSE mean (SD)	26.3 (2.4)* ^,^ ^#^	25.6 (2.9)* ^,^ ^#^	26.3 (2.4)* ^,^ ^#^	18.1 (3.4)	16.5 (6.3)
Clinical presentation	–	–	–	61.5% AD 38.5% PCA	84.6% AD 15.4% PCA
MCI stable/converted %	60/40%	26.7/73.3%	9.1/90.9%	–	–
Clinical presentation in MCI converted	20% AD 30% PCA 30% DLB 10% PSP 10% CBS	54.5% AD 18.2% PPA 9.1 PCA 9.1% bvFTD 9.1% Other	90% AD 10% bvFTD	–	–
Duration of follow‐up (Months) for stable MCI – mean (SD)	30.3 (18.1)	24 (13.8)	12 (−)^¥^	–	–
Time (Months) of conversion – mean (SD)	21.6 (17.7)	16.2 (11)	10.1 (4.6)	–	–

AD, Alzheimer's disease; bvFTD, behavioral variant of frontotemporal dementia; CBS, corticobasal syndrome; DLB, Dementia with Lewy body; PCA, posterior cortical atrophy; PSP, progressive supranuclear palsy; PPA, primary progressive aphasia; SD, standard deviation.

* Significantly different compared with Dementia A+T+ (*p* < 0.05 adjusted for multiple comparisons: Bonferroni);

^#^ Significantly different compared with Dementia A+T− (*p* < 0.05 adjusted for multiple comparisons: Bonferroni);

^¥^ Only one participant.

### Prognostic accuracy of CSF AT in MCI groups

The prognostic accuracy of AT biomarkers, as capability to identify MCI that will convert in dementia, was explored in the two extreme MCI groups (A−T− and A+T+).

**Figure 3:**
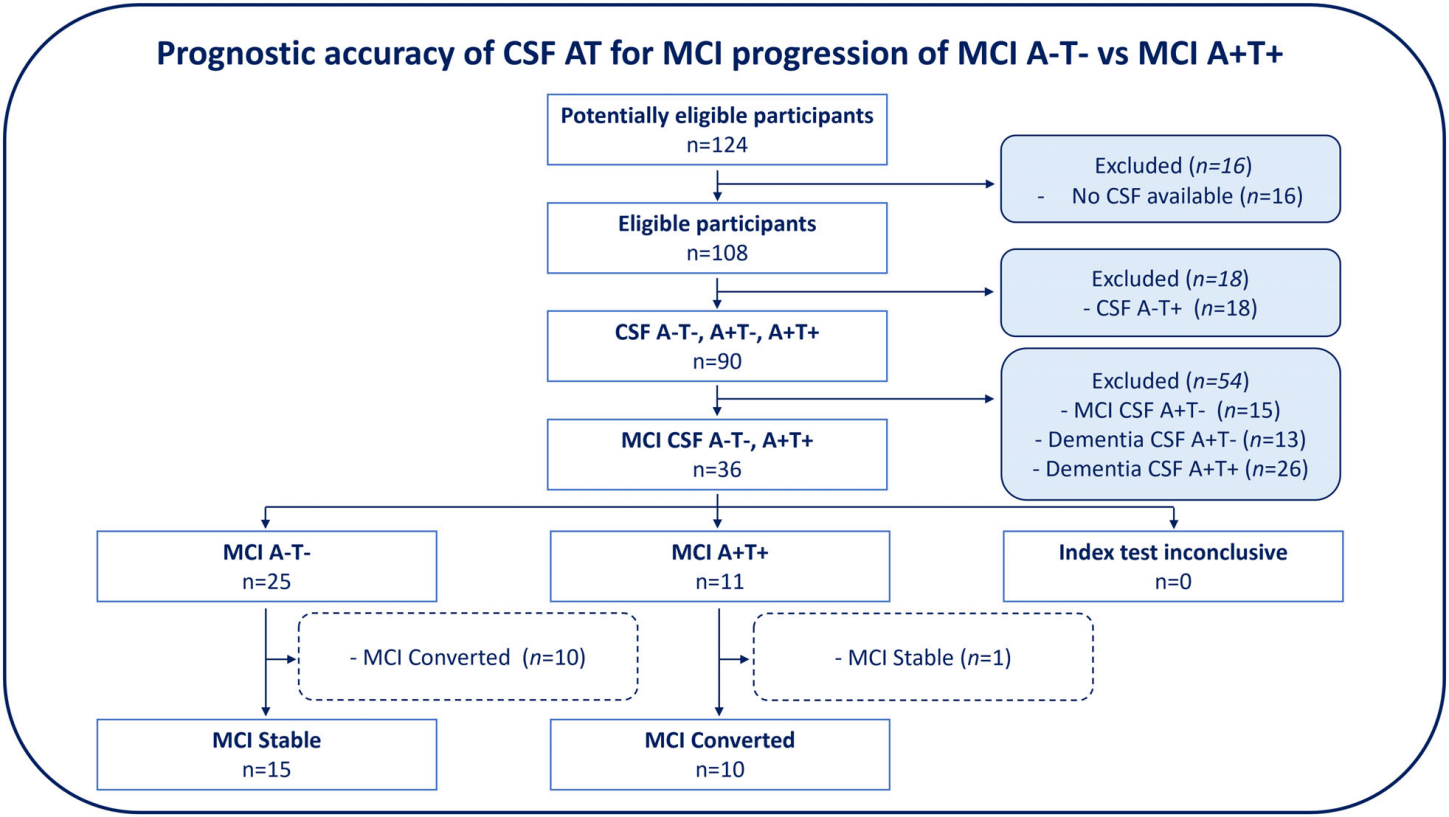
CSF markers: Amyloid (A) and Tau (T) flow diagram. Flow diagram shows performance testing structure of the CSF AT biomarkers. The test was conducted in the MCI A−T− and A+T+ groups, by comparing against the clinical status (MCI clinical status: Converted or Stable). The expected profile for MCI Converted is A+T+, and the expected profile for MCI Stable is A−T−. As seen in the figure, mixed AT profiles (A−T+, or A+T−) were excluded, in order to reduce the potential for including inaccurately classified cases.

In the MCI A−T− group, 15 participants (60%) remained stable and 10 (40%) converted to dementia. In the MCI A+T+ group, only one participant remained stable while all the other participants (10) converted to dementia (Fig. 3).

The mean of the duration of follow‐up was 32 months (range 12‐60 months), while the mean of the time of conversion to dementia was 14.3 months (range 3‐45 months) and the median was 12 months.

Investigating the rate of progression to dementia based on clinical classification, the AT biomarkers together showed a good positive predictive value with 90.91% of MCI A+T+ converting to dementia, but poor negative predictive value (Table 3) with 40% of MCI A−T− progressing to a mix of AD and non‐AD dementias.

**Table 3:** Prognostic accuracy for MCI progression of MCI A−T− vs MCI A+T+.

Diagnostic performance	Value	95% CI
Sensitivity	50.00%	27.20% to 72.80%
Specificity	93.75%	69.77% to 99.84%
Positive likelihood ratio	8.00	1.14 to 56.10
Negative likelihood ratio	0.53	0.34 to 0.84
Positive predictive value	90.91%	58.78% to 98.59%
Negative predictive value	60.00%	48.73% to 70.30%
Accuracy	69.44%	51.89% to 83.65%

Diagnostic indices based on CSF Aβ42 and pTau‐181 positivity in MCI (A−T− and A+T+) patients.

### Added value of N to CSF AT biomarkers

Investigating the additional contribution of neurodegenerative N measure to AT classification in MCI A−T− group, all micro‐ and macrostructural measures were included in ROC analyses. As summarized in Table 4, AngleR resulted the neurodegenerative measure with the higher area under the curve (0.800) in differentiating MCI A−T− stable and converted.

**Table 4:** Prognostic accuracy of MRI Neurodegenerative measures to differentiate MCI stable vs converting MCI A−T− patients.

(a) Neurodegenerative measure	AUC	Std. error	Asymptotic significance	95% CI lower bound	95% CI upper bound
***AngleR***	***0.800***	***0.092***	***0.013***	***0.619***	***0.981***
PerpPD^+^	0.587	0.131	0.471	0.330	0.844
ParlPD	0.387	0.114	0.346	0.163	0.610
FA	0.687	0.120	0.120	0.451	0.922
MD	0.573	0.100	0.542	0.322	0.825
Cortical GM fr	0.660	0.128	0.183	0.427	0.893
Cortical thickness	0.440	0.120	0.618	0.204	0.676
Hippocampal fr	0.607	0.116	0.375	0.380	0.834

(b) Diagnostic performance *AngleR*	Value	95% CI
Sensitivity	70.00%	34.75% to 93.33%
Specificity	86.67%	59.54% to 98.34%
Positive likelihood ratio	5.25	1.36 to 20.30
Negative likelihood ratio	0.35	0.13 to 0.91
Positive predictive value	77.78%	47.51% to 93.12%
Negative predictive value	81.25%	62.22% to 91.94%
Accuracy	80.00%	59.30% to 93.17%

The table (a) indicates the MCI A−T− stable vs converted classification performance of micro‐ and macrostructural measures. The performance was assessed by measuring area under the curve (AUC). Table (b) shows diagnostic indices based on adjusted AngleR values in MCI A−T− patients. Bold and italic values indicate as a stylistic choice to highlight the best result.

CI, confidence interval; Std, standard.

**Figure 4:**
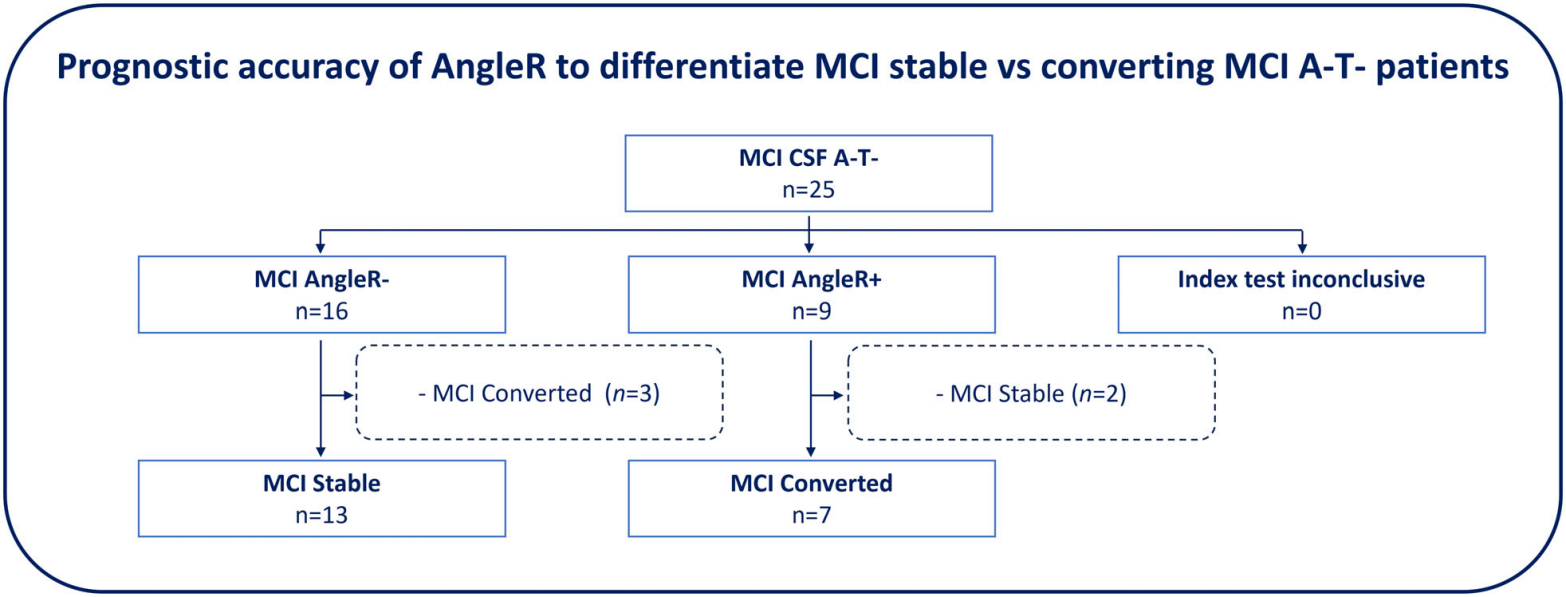
AngleR flow diagram in MCI A−T−. Flow diagram shows the performance testing structure of AngleR conducted in the MCI A−T− group, for comparison against the clinical profile (MCI clinical status: Converted or Stable). AngleR status, positive (+) or negative (−), was determined by the cutoff defined as the best point with the highest value obtained by averaging sensitivity and 1 − specificity.

To summarize the diagnostic power of AngleR, the accuracy (ACC), sensitivity (SENS), specificity (SPEC), positive likelihood ratio (LR+), negative likelihood ratio (LR−), positive predictive value (PPV), and negative predictive value (NPV) were computed at the best point along the ROC curve (Table 4). We defined the best point as the one with the highest value obtained by averaging sensitivity and 1 − specificity. The adjusted AngleR cutoff identify was −0.0137 (Fig. 4).

**Figure 5:**
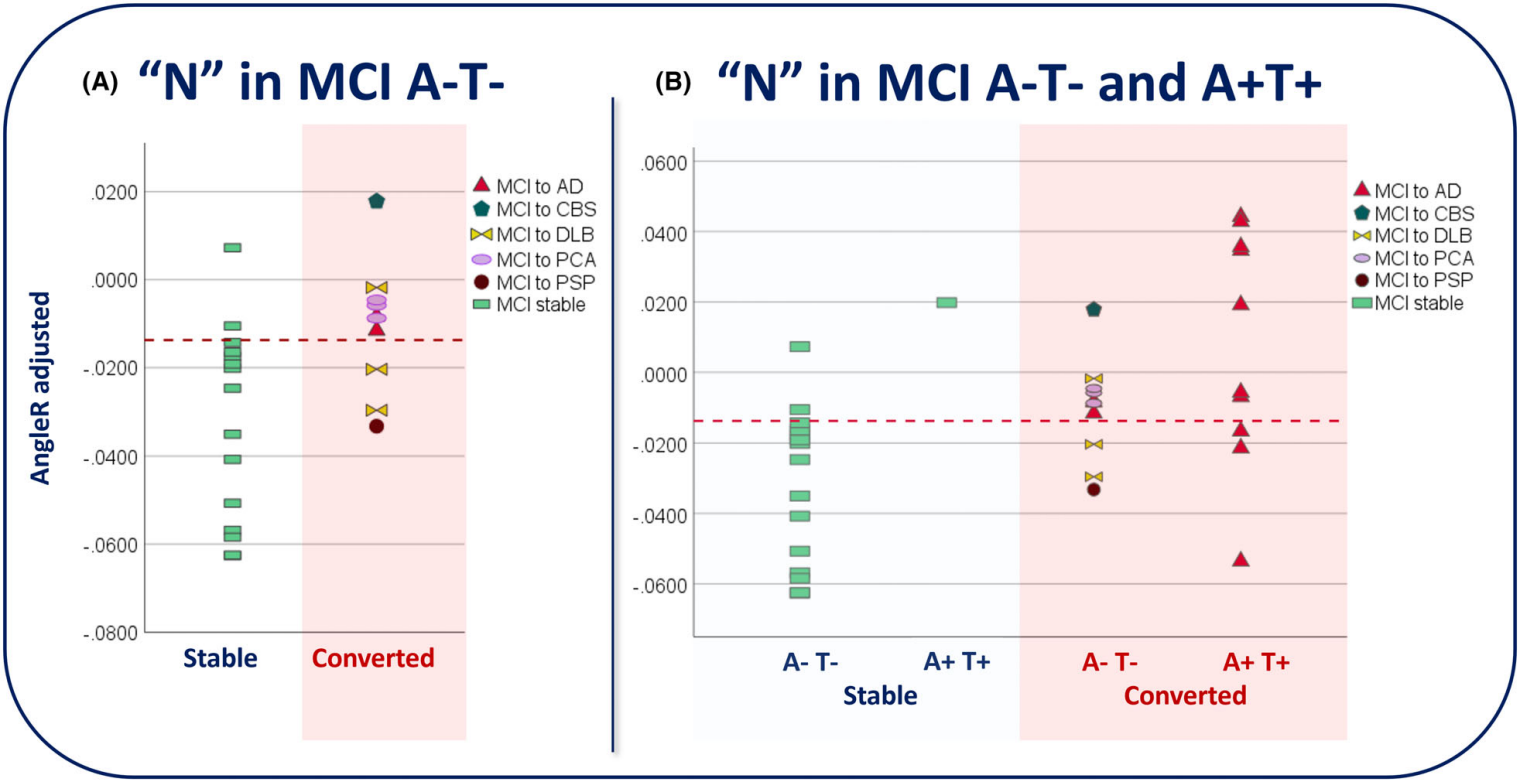
AngleR diagnostic power in MCI A−T− and MCI A+T+ stable vs converted. Panel (A) shows separation between stable and converted MCI A−T− using whole‐brain AngleR value. Panel (B) shows separation between stable and converted MCI A−T− and A+T+ using whole‐brain AngleR value. AD, Alzheimer's disease; CBS, corticobasal syndrome; DLB, Dementia with Lewy body; PCA, posterior cortical atrophy; PSP, progressive supranuclear palsy.

The separation between stable and converted MCI A−T− obtained using this threshold is visually summarized in Figure 5A.

Applying the AngleR threshold previously calculated to the other AT groups, Figure 5B displays the potential utility of the whole‐brain AngleR values to separate participants stable and converted to dementia.

To further investigate the improvement in differentiating stable and converted MCI groups adopting whole‐brain AngleR as additional N marker, the diagnostic performance of triple negative (A−T−N−) and triple positive (A+T+N+) MCI classification was tested. The same cutoff previously calculated within the MCI A−T− group was used.

Results showed that adopting AngleR as a marker of N led to a significant improvement in the prognostic accuracy of the combined ATN biomarkers compared with the AT biomarkers alone (Table 5). Though the PPV slightly decreased in the triple classification, the NPV was dramatically improved (+21.25%), determining a strong overall improvement in accuracy (+13.89%).

**Table 5:** Prognostic accuracy of ATN classification in stable vs converting MCI patients (A−T−N− and A+T+N+).

Diagnostic performance	AT value	ATN value	95% CI
Sensitivity	50.00%	**↑** 70.00%	34.75% to 93.33%
Specificity	93.75%	**↓** 92.86%	66.13% to 99.82%
Positive likelihood ratio	8	**↑** 9.8	1.42 to 67.64
Negative likelihood ratio	0.53	**↑** 0.32	0.12 to 0.84
Positive predictive value	90.91%	**↓** 87.50%	50.35% to 97.97%
Negative predictive value	60.00%	**↑** 81.25%	62.45% to 91.87%
Accuracy	69.44%	**↑** 83.33%	62.62% to 95.26%

This table shows the comparison between the diagnostic performance of AT classification (previously reported in Table 4) and that obtained using the ATN classification.

**↑** Improved compared with AT; worse compared with **↓** AT.

## Discussion

In the present study, our intention was to investigate the added value that cortical microstructural information from DTI can bring for improving classification provided by CSF Aβ42 and pTau‐181 markers. This additional information was found to be particularly valuable in one of the most problematic MCI groups from a clinical point of view, characterized by amyloid and pTau negative markers (MCI A−T−).

In clinical research, the ATN framework combined with clinical observation is a very promising approach to identify early patients with AD. However, in clinical practice, many patients can present cognitive symptoms overlapping with AD symptoms but due to a different neuropathological process. In clinically symptomatic patients with negative AD biomarkers, and in the absence of robust biomarkers to detect other neuropathological processes, it is a challenge for clinicians to decide the most appropriate diagnosis and treatment.

As is well known, protein biomarkers can be strongly related to specific neuropathological processes, so they show capability to detect the presence of the related pathology (i.e., high positive predictive value assessed against postmortem pathology). Furthermore, tau PET has shown to have good positive predictive value for clinical progression,^33^ although fluid biomarkers such as pTau‐181 have been found to be more closely linked to amyloid PET than tau PET,^34^ changing earlier in the disease course^35^ and consequently having less positive predictive value for near‐term clinical progression. However, in this study, CSF A+T+ status had relatively strong positive predictive value for progression to AD dementia. Even in cases with positive A and T biomarkers, there can be additional comorbidities and co‐pathologies, which one or more N biomarkers can help to shed further light upon.

Among the A−T− cases, there could be some biomarker false negatives, but even if the A and T biomarkers were perfect, there is clear added value of an N biomarker to relate to non‐AD neurodegeneration and clinical progression or lack thereof. As suggested by many previous lines of evidence^33^, ^36^, ^37^ neuropathological processes determine cortical or subcortical structural alterations in gray or white matter, so one of the main advantages of using neurodegenerative measures is its relative independence from the specific proteinopathy.

Despite all neurodegenerative processes causing specific structural alterations, by focusing attention on the cortical changes in the main dementia forms, it appears clear that detection of early cortical microstructural changes in MCI (especially MCI A−T−) can be particularly useful for clinically defined progression from MCI to dementia, supporting clinicians in the diagnostic process.

In the last few years, cortical diffusivity methods^13^, ^16^, ^17^ have gained traction in the field of cortical investigations where they were mainly used to investigate cortical microstructural changes in neurodegenerative disorders. A recent study that has utilized cohorts with autopsy confirmation has shown that cortical diffusivity measures are associated with the major neuropathological hallmarks of AD.^15^

From a diagnostic point of view, cortical microstructural measures have been used in different neurological conditions, for example, to differentiate AD and controls, AD and posterior cortical atrophy^38^ or different forms of fronto temporal dementia.^39^, ^40^, ^41^

In this study, we investigated patients with MCI or dementias, classified based on CSF amyloid and pTau biomarkers, and restricted to the amyloid continuum (i.e., A−T−, A+T−, and A+T+, excluding A−T+). All the micro‐ and macrostructural measures showed a progression of severity along the amyloid continuum confirming the sensitivity to AD progression previously showed by other studies.^14^, ^16^ These studies displayed biphasic trajectories, in which transient changes in the early stages of the AD progression (cognitively normal, amyloid positive) run directionally counter to the effects of neurodegeneration in more severe disease. Inflammatory and neurodegenerative processes may be contributing to the biphasic pattern determining the early decreases in microstructural signal in cognitively unimpaired groups, followed by progressive signal increases during the symptomatic phases (MCI, dementia), mainly due to neuronal loss and minicolumnar disruption. Among the microstructural measures, AngleR alone exhibited significant differences for both A+ groups compared with A−T−.

In the second part of the study, the participants were classified into five different groups based on clinical syndromic status (MCI or dementia) and biomarker status (AT). To investigate the diagnostic power of CSF AT markers to identify MCI patients converting to dementia, we compared the two extreme MCI groups (MCI A−T− vs MCI A+T+). The MCI A+T− group was not included in this investigation because being an intermediate state between MCI extremes in a hypothetical continuum was not suitable for the purpose of the analysis. As shown by the conventional diagnostic indices (sensitivity, specificity, accuracy, positive and negative likelihood, positive and negative predictive values) calculated using CSF A and T cutoffs, consistent with previous studies,^42^, ^43^ CSF AT markers showed a very high positive predictive value (90.91%) and a lower negative predictive value (60%). Analyzing the meaning of the low negative predictive values, we observed that only the 20% of MCI A−T− converted were potentially “false negative” (having converted to AD) while the remaining 80% included patients converted to non‐AD dementia (30% Lewy body dementia, 30% posterior cortical atrophy, 10% progressive supranuclear palsy, and 10% corticobasal syndrome). However, without CSF follow‐up and/or autopsy confirmation, we cannot conclusively consider the MCI A−T− converted to AD as “pure” false negative, because the apparent AD presentation may be due to other neuropathologic processes with overlapping clinical presentation (e.g., TDP‐43).

In order to investigate the potential utility of N measures to improve the negative predictive values and, in general, the accuracy, a ROC analysis on MCI A−T− was performed, including all the micro‐ and macrostructural MRI measures previously investigated. The results revealed that AngleR was the N measure with the highest AUC and good positive and negative predictive values (PPV = 77.78%; NPV = 81.25%).

Recent evidence^44^, ^45^ showed that different proposed markers of neurodegeneration (e.g., NfL, hippocampal volume, cortical thickness, FDG PET SUVR, and CSF t‐tau) are poorly correlated at all disease stages, resulting in substantial misclassification.

Among the candidates' biomarkers of neurodegeneration, NfL is a promising biomarker for rapid screening to identify or reject neurodegeneration.^46^ However, NfL does not provide topographical information and provides limited information for separating specific disorders of cognitive impairment (e.g., FTD vs AD), prodromal (e.g., CU vs subjective cognitive decline or MCI), or preclinical conditions (e.g., CU Aβ − vs CU Aβ+).^47^

Our findings suggest that simple whole‐brain cortical microstructural measures bring added value by improving classification and providing new metrics complementary to CSF Aβ42 and pTau‐181. Moreover, as shown by recent studies,^14^, ^15^, ^16^, ^38^, ^39^, ^40^, ^41^ regional cortical microstructural measures providing a topographical visualization of cortical damage and disease spreading may be particularly useful for disease staging and differential diagnosis. The topographical distribution of the cortical degeneration provided by microstructural measures can compensate for the main limitation of NfL (lack of spatial information). Therefore, cortical microstructural measures, in combination with NfL, or alone, may represent a very promising noninvasive and cost‐effective index of neurodegeneration. Further work will investigate the clinical utility of regional cortical microstructural measures in real‐word clinical settings.

Finally, we calculated the improvement due to adopting AngleR as N measure in the ATN classification of MCI patients (A−T−, A+T+) stable and converted. Results showed that the NPV was dramatically improved (+21.25%), while the PPV was little changed (slight decrease −3.41%), resulting in a strong accuracy improvement (+13.89%).

These findings indicate the potential clinical utility of novel cortical diffusivity measures (e.g., AngleR) to support correct classification of patients, identifying cortical alterations even in A−T− patients.

The study has some limitations. The first limitation is the sample size; larger MCI samples may be required to investigate the diagnostic accuracy of cortical diffusivity measures. The findings of the study are cross‐sectional, and future work with longitudinal imaging data is needed to evaluate measures for tracking disease progression. This study focused on the amyloid continuum, including in the study patients with (A+T− A+T+) and without Aβ42 or pTau positivity (A−T−). Further work will investigate more complexities of the real‐world clinical setting, including MCI A−T+, Dementia A−T−, and Dementia A−T+ groups.

MRI scans were acquired in the same center using two different acquisition protocols. Although the differences in protocols were included in the analyses, this may have had an effect on the values. Another limitation is the different duration of the clinical data available for each patient. It is important to highlight that data used in the present study are from a clinical setting, and therefore, different from data acquired within research projects, the patients were not clinically evaluated for the same period. This is not a complication for the converted MCI, because all the diagnoses after conversion were confirmed at follow‐up, but could be a limitation for the MCI stable group. The mean of the stability period observed was reasonable (32 months), a few participants had only 12 months follow‐up information, and therefore, it is possible that some of these participants could have converted a few months later.

In this study, we used only whole‐brain measures that may not always be sensitive to more focal cortical alterations and therefore not provide optimal detection of dementia forms not primarily characterized by cortical alterations (e.g., PSP). Future work is needed to assess the diagnostic power of disease‐specific regional cortical signatures.

Co‐existent neuropathological processes were not investigated. Further studies with autopsy confirmed cohorts may help to investigate the potential impact of comorbidities on MRI values and clinical progression.

Finally, we did not have data from some other N marker candidates to perform a head‐to‐head comparison (e.g., NfL). This comparison could be interesting although the two kinds of markers (cortical diffusivity and NfL) provide different and complementary information (NfL informs about axonal degeneration while cortical diffusivity measures inform cortical microstructural changes and can also provide the spatial pattern of damage).

## Conclusion

New cortical diffusion measures of neurodegeneration may be integrated with fluid AT markers to improve confidence for the clinician to recommend decisions about patient management.

## Author Contributions

The authors confirm contribution to the paper as follows: Study conception and design: MT, GRR, and SAC; acquisition and analysis of data: MT, GF, GRR, VEC, ES, IH, DG, and AA; drafting manuscript: MT, GF, GRR, VEC, ES, DG, SAC, and AA. All authors reviewed the results and approved the final version of the manuscript.

## Conflict of Interest

S.A. Chance is a co‐founder of a company, Oxford Brain Diagnostics, from which he has received funding for the research and preparation of this manuscript; M. Torso, G.R. Ridgway, and I. Hardingham are currently employed at a company, Oxford Brain Diagnostics; S.A. Chance has a patent (WO2016162682A1) related to the diffusion MRI analysis used in the present study; G. Fumagalli, V.E. Contarino, E. Scarpini, D. Galimberti, and A. Arighi report no disclosures relevant to the manuscript.

## Supporting information


Table S1.

## Data Availability

Anonymized data can be obtained by reasonable request from any qualified investigator.
