# Supplementary material for: Clinical utility of diffusion MRI‐derived measures of cortical microstructure in a real‐world memory clinic setting
Source: Ann Clin Transl Neurol. 2024 Jul 24;11(8):1964–76. doi: 10.1002/acn3.52097 (PMC11330221; doi:10.1002/acn3.52097)
Supplement: Supplementary file 1 — Table S1. [file ACN3-11-1964-s001.docx]

**Supplemental materials**

**Table 1 Micro and Macrostructural MRI values**

| ***Microstructural*** | ***Group*** | ***Mean (SD)*** | ***F*** | | ***p-value*** | **η_p_^2^** |
| --- | --- | --- | --- | --- | --- | --- |
| ***AngleR*** | *A-T-* | *0.841 (0.025) *#* | *9.270* | | *<0.001* | *0.196* |
|  | *A+T-* | *0.869 (0.031)* |  |  |  |  |
|  | *A+T+* | *0.883 (0.034)* |  |  |  |  |
| ***PerpPD^+^*** | *A-T-* | *1.33 (0.083) #* | *5.950* | | *<0.005* | *0.135* |
|  | *A+T-* | *1.48 (0.168)* |  |  |  |  |
|  | *A+T+* | *1.57 (0.286)* |  |  |  |  |
| ***ParlPD*** | *A-T-* | *0.442 (0.017) #* | *3.894* | | *0.025* | *0.093* |
|  | *A+T-* | *0.462 (0.029)* |  |  |  |  |
|  | *A+T+* | *0.476 (0.052)* |  |  |  |  |
|  | *A-T-* | *0.159 (0.010)* |  | |  |  |
| ***FA*** | *A+T-* | *0.157 (0.012)* | *0.467* | | *n.s* | *0.012* |
|  | *A+T+* | *0.158 (0.020)* |  | |  |  |
| ***MD*** | *A-T-* | *0.00114 (0.00016)* | *2.591* | | *n.s* | *0.064* |
|  | *A+T-* | *0.00125 (0.00021)* |  |  |  |  |
|  | *A+T+* | *0.00123 (0.00020)* |  |  |  |  |
| ***Macrostructural*** | ***Group*** | ***Mean (SD)*** | | ***F*** | ***p-value*** | **η_p_^2^** |
| ***Cortical volume fraction*** | *A-T-* | *0.2724 (0.024) *#* | | *10.645* | *<0.001* | *0.219* |
|  | *A+T-* | *0.2498 (0.018)* | |  |  |  |
|  | *A+T+* | *0.2479 (0.018)* | |  |  |  |
| ***Cortical thickness*** | *A-T-* | *0.244 (0.093) #* | | *8.030* | *<0.001* | *0.174* |
|  | *A+T-* | *0.233 (0.145)* | |  |  |  |
|  | *A+T+* | *0.224 (0.181)* | |  |  |  |
| ***Hippocampal volume fraction*** | *A-T-* | *0.00481 (0.00073) *#* | | *10.624* | *<0.001* | *0.218* |
|  | *A+T-* | *0.00425 (0.00066)* | |  |  |  |
|  | *A+T+* | *0.00405 (0.00052)* | |  |  |  |

η_p_^2^ = partial eta squared; *Significantly different compared to A+T-; ^#^Significantly different compared to A+T+. *^#^ p < 0.05 adjusted for multiple comparisons: Bonferroni.
